# Little or no gene flow despite F_1_ hybrids at two interspecific contact zones

**DOI:** 10.1002/ece3.1942

**Published:** 2016-03-09

**Authors:** Natasha E. Mckean, Steven A. Trewick, Mary Morgan‐Richards

**Affiliations:** ^1^ Ecology GroupInstitute of Agriculture and EnvironmentMassey UniversityPalmerston NorthNew Zealand

**Keywords:** Divergence, *Hemideina*, hybridization, introgression, population genetics

## Abstract

Hybridization can create the selective force that promotes assortative mating but hybridization can also select for increased hybrid fitness. Gene flow resulting from hybridization can increase genetic diversity but also reduce distinctiveness. Thus the formation of hybrids has important implications for long‐term species coexistence. This study compares the interaction between the tree wētā *Hemideina thoracica* and its two neighboring species; *H. crassidens* and *H. trewicki*. We examined the ratio of parent and hybrid forms in natural areas of sympatry. Individuals with intermediate phenotype were confirmed as first generation hybrids using nine independent genetic markers. Evidence of gene flow from successful hybridization was sought from the distribution of morphological and genetic characters. Both species pairs appear to be largely retaining their own identity where they live in sympatry, each with a distinct karyotype. *Hemideina thoracica* and *H. trewicki* are probably reproductively isolated, with sterile F_1_ hybrids. This species pair shows evidence of niche differences with adult size and timing of maturity differing where *Hemideina thoracica* is sympatric with *H. trewicki*. In contrast, evidence of a low level of introgression was detected in phenotypes and genotypes where *H. thoracica* and *H. crassidens* are sympatric. We found no evidence of size divergence although color traits in combination with hind tibia spines reliably distinguish the two species. This species pair show a bimodal hybrid zone in the absence of assortative mating and possible sexual exclusion by *H. thoracica* males in the formation of F_1_ hybrids.

## Introduction

Hybridization is the production of offspring between genetically distinct individuals (Harrison 1993), and the fitness of hybrids has fundamental implications for the populations involved. The fitness of F_1_ hybrids produced from genetically distinct populations will determine the extent of gene flow and strength of selection (Butlin 1987). Even limited fertility can result in introgression and allow passage of adaptive alleles between groups of individuals that are considered to be distinct species (Anderson et al. 2009; Song et al. 2011). Fertile hybrids enable gene flow among sympatric populations that might eventually result in loss of phenotypic and genetic distinctiveness. However, hybrids with lower fitness than parental taxa can cause increased reproductive interference and their production will be costly to individuals. Thus, where hybrids are sterile or have reduced viability and/or fertility any trait that reduces interspecific mating (reinforcement) could be at a selective advantage. Hybrid sterility will favor reproductive character displacement that might be detected in regions of sympatry (Dieckmann and Doebeli 1999). However, where fertile hybrids create gene flow, three factors will reduce the probability of reinforcement; (1) recombination among genes influencing hybrid fitness and between genes for assortative mating, (2) gene flow from outside the contact zone, and (3) stabilizing selection on the mate recognition system (Butlin 1987). The form that hybrid zones take is influenced by these outcomes and can range from bimodal, where parental forms predominate and individuals of mixed ancestry are few, to unimodal, where individuals of mixed ancestry predominate in areas of sympatry (Jiggins and Mallet 2000). For species that exchange alleles, selection can favor an increase or a decrease in reproductive isolation depending on the relative fitness advantages to the individual, and the initial allelic variation existing within the populations. Gene flow is likely to reduce genetic distinctiveness and thus increase competitive interactions, while diverging selection would favor traits that reduce competition. However, an equilibrium might develop where different factors have opposing outcomes, resulting in a stable tension zone (Key 1968; Barton and Gale 1993).

Studying patterns of gene flow therefore provide valuable insights into the nature of species and speciation (Barton and Hewitt 1985; Abbott et al. 2013). In New Zealand, species of endemic Orthoptera known as tree wētā have predominantly parapatric ranges (Gibbs 2001). However, at their range margins, there are areas of sympatry of varying size where the opportunity for gene flow between species exists. Field observations reveal that members of different species that are characterized by distinct morphology, genetics and cytogenetics (Morgan‐Richards and Townsend 1995; Morgan‐Richards 1995; Morgan‐Richards and Gibbs, 2001) are willing to cohabit in daytime refuge holes in trees (Trewick and Morgan‐Richards 1995, 2000). Their reproductive interactions are not readily detected as all activity is nocturnal.

**Figure 1:**
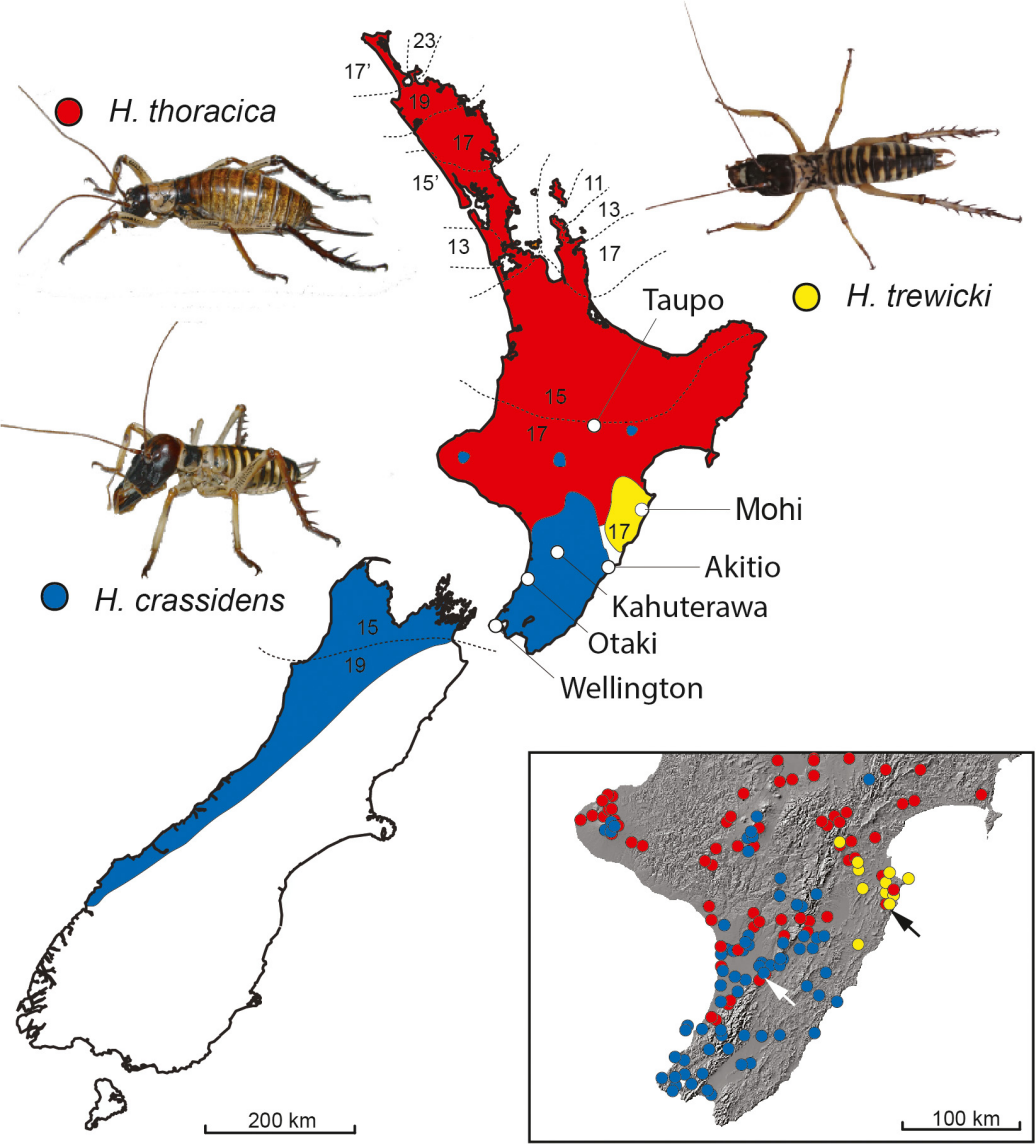
(A) Distribution of three New Zealand species of tree wētā (*Hemideina*). Distributions of chromosome races within species are delineated by dotted lines, with number representing chromosome number of males (XO). The distributions of the *H. thoracica* and *H. crassidens* chromosome races were taken from Morgan‐Richards and Wallis (2003) and Morgan‐Richards (2000) respectively. Locations of wētā sampling are indicated. Inset shows mosaic nature of tree wētā species distribution in the southern North Island and two sampled sympatric sites indicated with arrows.

In North Island New Zealand (Fig. 1), three species of *Hemideina* tree wētā occur naturally. The 17‐chromosome race of the tree wētā *Hemideina thoracica* makes contact with two sister species; *H. crassidens* (2n = 15 (XO)) and *H. trewicki* (2n = 17 (XO); Morgan‐Richards 1997; Bulgarella et al. 2014). Both interspecific contact regions are at the southern limits of the *H. thoracica* range (Fig. 1). In the central part of the *H. thoracica* range, the 17‐chromosome race meets conspecifics with 15 chromosomes at Lake Taupo, and concordance of genetic trait clines suggests a semipermeable barrier to gene flow and the wētā are morphologically indistinguishable (Morgan‐Richards et al. 2000). In contrast, where *H. thoracica* is sympatric with either *H. crassidens* or *H. trewicki* the wētā species are morphologically distinct and limited genetic data suggests reproductive isolation of species (Morgan‐Richards 1995; Morgan‐Richards et al. 1995; Bulgarella et al. 2014). However, in these narrow regions of sympatry, cohabiting of different species in the same daytime refugia is common, suggesting a lack of species specific mate recognition. Rare individuals of intermediate color pattern have been found but whether these were interspecific hybrids and whether they were fertile could not be determined in the field. If they are hybrids and are capable of backcrossing with parental species introgression would result, and it is possible that additional hybrids could be morphologically cryptic.

We use a combination of morphology, cytogenetics, and population genetics to establish the identity of hybrid tree wētā at two areas of sympatry and assess the extent of gene flow among species. Hybrid disadvantage is expected to select for greater divergence of species in sympatry when compared to allopatric populations. Thus we sought evidence of divergence in timing of development by comparing proportion of adults/nonadults of each species at one sampling event in sympatry. We compared average size of adult females in allopatric and sympatric populations. If hybrids are rare or/and infertile then a bimodal pattern of phenotypes and genotypes would be expected. However, phenotype might hide cryptic hybrids and introgression so we investigated these possibilities using cytogenetics, mtDNA, and nuclear markers.

## Material and Methods

### Study sites

Suitable study sites were identified using the ratio of each species at locations to determine they truly represented areas of sympatry. Previous studies had identified a mosaic pattern of micro‐allopatry through much of southern North Island, where the majority of sites were home to just *H. thoracica* or just *H. crassidens* (Fig. 1 inset). Competitive interactions are thought to produce the current distribution (Bulgarella et al. 2014), however, local sympatry has been detected at a few locations. We estimated the number of individuals with parental phenotypes relative to individuals with intermediate (hybrid) phenotype within two contact regions. We compared the ratio of adults to juveniles in population samples where the species were sympatric, as differences in timing of maturity could reproductively isolate populations.

At the southernmost region of the *H. thoracica* distribution, an area of sympatry between *H. thoracica* and *H. crassidens* (Kahuterawa)*,* and one between *H. thoracica* and *H. trewicki* (Mohi) were sampled (Fig. 1). Allopatric populations of *H. thoracica* and *H. crassidens* were sampled to look for evidence of character displacement by comparing traits of allopatric and sympatric populations and to evaluate interspecific similarity of genetic markers resulting from ancestral polymorphism rather than introgression (Fig. 1). Allopatric population samples were geographically separate from the contact areas while still belonging to the same chromosome race (Morgan‐Richards 1997; Bulgarella et al. 2014). *Hemideina trewicki* has a narrow distribution that might overlap with *H. thoracica* or *H. crassidens* throughout most of its range (Trewick and Morgan‐Richards 1995), thus we did not sample allopatric populations of this species.

### Ratio of parent phenotypes to hybrid phenotypes

At Mohi Bush, Hawkes Bay (S 39.45, E 176.88333) where *H. thoracica* and *H. trewicki* are sympatric (Morgan‐Richards 1995) a sample of 101 tree wētā was collected. In the Kahutawera Valley Manawatu (S 40.47184, E 175.60943) where *H. thoracica* and *H. crassidens* occur (Bulgarella et al. 2014), a similar sample of 105 wētā was collected. Wētā collection within the New Zealand conservation estate was done under permit from the New Zealand Department of Conservation (TW‐32116‐FAU; W/E 31465/FAU).

Surveys involved locating suitable roost holes in dry dead wood and extracting any wētā inside. Species identification used color and pattern of the abdomen and thorax (Ramsey and Bigelow 1978; Morgan‐Richards 1995) and the number of prolateral hind tibial spines (Table 1). Tree wētā younger than the fourth instar were not included because color characteristics are difficult to determine in small individuals. Wētā identified as putative hybrids were those that had intermediate abdominal coloration with only light banding, which did not closely resemble either of the parent species. Wētā were released after identification, with the exception of a random sample of the parental individuals and all putative hybrids that were retained for genetic analyses. Species observations were used to calculate frequency of the parent species and hybrids, and compared to Hardy**–**Weinberg (HW) expectations if the populations were freely interbreeding (but producing just parental and F_1_ offspring) using a *χ* ^2^ test. The proportion of adults and juveniles between the population samples collected in sympatry was compared with a two‐tailed Fisher's exact test.

**Table 1:** Combination of phenotypic traits that distinguish three North Island New Zealand tree wētā species (genus *Hemideina*), and their frequency in allopatric and sympatric populations and putative interspecific hybrids

Species	Location	Sample size	Abdominal Color			Pronotum		Mesonotum		Metanotum		Abdominal Bands			Dorsal Stripe			Hind tibia spines				
			yellow	orange	brown	Dark	Pale	Dark	Pale	Banded	Dark	Yes	Faint	No	Yes	Dashed	No	6	6.5	7	7.5	8
*Hemideina crassidens*	Wellington	26	26			26			26	26		26			26							26
*Hemideina crassidens*	Kahuterawa	26	26			25	1		26	26		26			26					2		24
Putative hybrid	Kahuterawa	9		9			9	9			9		9			9		1	1	5	1	1
*Hemideina thoracica*	Kahuterawa	22			22		22	22			22			22			22	21	1			
*Hemideina thoracica*	Taupo	22			22		22	22			22			22			22	17		5		
*Hemideina thoracica*	Mohi	26			26		26	26			26			26			26	26				
Putative hybrid	Mohi	1		1			1	1			1		1			1						1
*Hemideina trewicki*	Mohi	27	27				27		27	27		27			27							27

### Phenotype

For color characters and tibial spine counts, 159 tree wētā (both sexes) were studied (Table 1). Comparative material came from allopatric populations in Wellington (*H. crassidens*) and near Lake Taupo (*H. thoracica*; Fig 1). The coloration and banding pattern of the pronotum, mesonotum, and metanotum, presence of a dorsal stripe on the abdomen, and the number of prolateral spines on each hind tibia, were recorded for each individual. Tubercles (small protuberances) in the position usually occupied by prolateral spine IV in *H. crassidens* and *H. trewicki* were recorded as half spines.

Evidence for the divergence of traits in regions of sympatry was sought by comparing the size of adults from allopatric and sympatric populations. Character displacement theory predicts greater differentiation in sympatry than in allopatry. Length of the left hind tibia was measured using electronic callipers accurate to 0.01 mm, as an indicator of overall body size (Minards et al. 2014). ANOVA was performed with Tukey's test to check for significant differences between population means using Minitab 16 Statistical software. The size comparison used a sample of 65 adult females, because male tree wētā can mature at three different instars whereas females mature only at the tenth (Kelly and Adams 2010; Minards et al. 2014).

### Cytogenetics

Wētā were karyotyped as previously described (McKean et al. 2015). Both species pairs contain differences in the relative sizes of their chromosomes allowing differentiation of the three species (McKean et al. 2015). The karyotypes of F_1_ hybrids were predicted based on parental karyotypes and compared to karyotypes obtained from putative hybrids. For the Mohi species pair (*H. thoracica* and *H. trewicki*), total chromosome number is the same, so F_1_ hybrids would have 17 or 18 chromosomes (wētā have an XO sex‐determination system; Morgan‐Richards 1997). For the Kahuterawa species pair (*H. thoracica* and *H. crassidens*), the total chromosome number differs; thus hybrids were expected to have 2n = 16 (XO) or 17 (XX).

### DNA sequence data

For genotyping, a total of 107 wētā were studied from seven populations, including 10 putative hybrid wētā (Table 2). Wētā specimens were stored in 99% ethanol prior to DNA extraction using a standard salting out method (Sunnucks and Hales 1996). A portion of the cytochrome oxidase 1 (COI) mtDNA gene was amplified using a combination of PCR primers (Bulgarella et al. 2014). Fragments of two coding nuclear loci, sperm flagallar protein (SPEF) and testis kinase 1 (TESK1), were amplified and sequenced using primers developed for these genes in tree wētā by Twort (2012) and details provided in Table 3. As these three loci are coding, single nucleotide polymorphisms are not necessarily neutral, but no evidence of positive or constraining selection at either nuclear locus has been found (Twort 2012).

**Table 2:** Population samples of tree wētā (genus *Hemideina*) from North Island New Zealand were genotyped for 10 markers to provide evidence of species specific alleles and allow gene flow to be estimated. Karyotype and mitochondrial data were collected from a subset of the total sample

			*H. crassidens*				Putative hybrid	*H. thoracica*			Putative hybrid	*H. trewicki*
			Wellington	Akito	Otaki	Kahuterawa	Kahuterawa	Kahuterawa	Taupo	Mohi	Mohi	Mohi
		*n*	10	10	10	23	9	22	10	12	1	10
mtDNA	*H. thoracica*	A					1	5				
	*H. thoracica*	B								8		
	*H. thoracica*	a						11	10			
	*H. trew*	C										6
	*H. trewicki*	D										2
	*H. crassidens*	E				4						
	*H. crassidens*	F				1						
	*H. crassidens*	G				3						
	*H. crassidens*	H				2	1					
	*H. crassidens*	I				2	2					
	*H. crassidens*	J					1					
	*H. crassidens*	K					1					
	*H. crassidens*	L					1					
	*H. crassidens*	M					1					
	*H. crassidens*	a	10	10	10	10	1					
	Karyotype	*crass*	10			11	0.5					
		*thor*					0.5	10	10	12	0.5	
		*trew*									0.5	10
SPEF	Alleles	A									1	19
		B		1	14	25	5					1
		C					1	3	3	11		
		D		10		11	11	36	17	10	1	
		E						3		3		
		F		9	6	2	1					
		G				4						
		H				1						
TESK1	Alleles	A					9	36	2	17	1	19
		B										1
		C							17	6	1	
		D		17	13	34	9					
		E						2				
		F						1				
		G		3	5	9						
		H							1			
		I						1				
		J				3						
		K						1		1		
HR12	Alleles	166						1				
		174								1		
		179		4	5	2						
		184		15	15	42	9					2
		186				1	8	39	19	19	1	
		187		1		1						
		188						2	1	4		
		189					1					
		196									1	18
HR13A	Alleles	156						1				
		162				2						3
		166			4							
		168				23	4					
		170		16							1	17
		172					1	4				
		174					5	19	6	19		
		176								1		
		177		4			8	16				
		179						3		4	1	
		182							14			
		184						1				
		187			10	2						
HR35	Alleles	224		7	6	16	3					
		227			1	6						
		228				2						
		230				3						
		231		3								
		233		2		1	1					1
		235										2
		236			2							
		239					1					
		240		1	6	1						
		242		1	1	2	3	9				
		244		1	1	3	4	28	15	24	1	8
		246					1					
		247			2	2	2	5	5			1
		250		1		1	1	2			1	
		253		4		1						2
		254				1						
		255										3
		258				3						1
		260					1					
		262			1	1						
		265				1						
		270					1					
		274				1						
Hma04	Alleles	80					9	44	20	24	1	
		88		19	16	44	9				1	16
		90			4							4
		92				2						
		95		1								
										
HR14	Alleles	166		8								
		168			2	40	5					7
		183		12	18	4	13	42	20	24	2	13
HR43	Alleles	103		20								
		114			17	30	16	44	20	24	2	20
		125				1						
		127			3	15	2					

a Data obtained in previous studies (Morgan‐Richards et al. 2000; Bulgarella et al. 2014).

**Table 3:** Primers and their annealing temperatures used to amplify three loci for population genetic analysis of tree wētā (genus *Hemideina*) from North Island New Zealand

Locus		Primer‐forward		Primer‐reverse	Annealing temp. °C
Sperm flagallar protein	SPEF‐f	TCG CCA GTT CAG ACC TAG GAT GAGG	SPEF‐r	TGG CTC TGT ACA AGG CTG GGA	59
Testis kinase 1	TESK1‐f	CGG AAG TAG TAA GTG GGA CGC CG	TESK1‐r	CGC TGG TTG ACA TTG GAG TGG GA	67
Cytochrome oxidase I	Mtd10crass–f	AAC ACT TAT TTT GAT TCT TTG G	12weta‐r	ATT GCA CTT ATC TGC CAT ATT AG	53
Cytochrome oxidase I	1490thor–f	AAC TAA TCA CAA GGA TAT TGG	12weta‐r	ATT GCA CTT ATC TGC CAT ATT AG	54

PCR products were sequenced using a capillary ABI3730 Genetic Analyzer (Applied Biosystems Foster City, CA. USA). DNA sequences were visualized and aligned in Geneious v6.1.7 (http://www.geneious.com; Kearse et al. 2012). For mtDNA haplotypes we used the integer neighbor‐joining method (French et al. 2013) with reticulation tolerance set to zero. This allowed unequivocal assignment of each haplotype to species clusters. Variation at nuclear loci was visualized using minimum spanning networks (Bandelt et al. 1999). Networks were generated using PopART (Population Analysis with Reticulate Trees; Leigh and Bryant 2015). We used Fisher's exact test to determine whether the hybrid mitochondrial haplotypes suggested a species bias of mothers. Sequences are available at http://evolves.massey.ac.nz/DNA_Toolkit.htm.

### Microsatellite loci

Sixteen microsatellite primer pairs developed for South Island species of tree wētā were trialled. Six loci that consistently amplified in all three North Island species were used to genotype all specimens in this study. Amplification of DNA followed conditions used by King et al. (1998) and Hale et al. (2010). PCR products were pooled for genotyping on an ABI3730 Genetic Analyzer. The microsat plugin in Geneious v6.1.7 was used to score alleles. Fixed allelic differences between parent populations were identified, and putative F_1_ hybrids were expected to be heterozygote at these loci.

Micro‐Checker v2.2.3 (Van Oosterhout et al. 2004) was used to identify any scoring errors, the presence of null alleles, and large allele dropout. Any locus that presented problems in one but not the other two species was excluded for comparison for the problematic species only. As problems detected by Micro‐Checker may be due to the small size of some samples, this was taken into consideration before discarding data.

The two sequenced nuclear genes were coded as alleles so that they could be included in genotype analysis with the microsatellite data. Evidence of linkage disequilibrium among loci was sought with contingency tables analyzed with the Markov chain method to estimate expected *P*‐values (Raymond and Rousset 1995a) using Genepop v3.4 (Raymond and Rousset 1995b) online. All pairs of loci were tested, using genotypes from a single population and a single species, with 10,000 dememorisations and iterations, and with 1000 batches.

### Population genetic structure

We used the fixation index *F*_ST_ to seek evidence of reproductive isolation between populations. Populations of different species were expected to have low or no interbreeding, while populations of the same species at different locations might show some differentiation due to isolation by distance (Slatkin 1995). Pairwise *F*_ST_ values were calculated using Arlequin v3.5.1.3 (Excoffier and Lischer 2010) combining eight nuclear loci.

Genetic evidence was used to determine whether individuals identified as putative hybrids were hybrids and their likely category (F_1_, F_2_, backcross). Evidence of backcross or F_2_ hybrids would reveal that F_1_ hybrids were not infertile. To estimate the posterior probability that individual genotypes apportion to predefined parent species (1, 2), hybrid (F_1_) or backcross (B_1_, B_2_) classes we used NewHybrids v1.1 (Anderson and Thompson 2002). Later generation backcrosses (B_2_) were included for the *H. thoracica* and *H. crassidens* data set*,* but did not appear to influence the results for *H. thoracica* and *H. trewicki* so were removed for the analyses of this pair. Allopatric populations of *H. thoracica* and *H. crassidens* were included and labeled as such, but all Kahuterawa and Mohi individuals including F_1_ hybrids were left unidentified for their respective runs. For the Mohi wētā no allopatric populations were added as original results were straightforward. Multiple runs of the software with different random starting seeds always converged on the same pattern, so multiple runs were not averaged. We used 1,000,000 MCMC iterations and removed 10,000 initial iterations as burnin.

To infer population structure using individual wētā genotypes we used the Bayesian assignment approach of Structure v2.3.4 (Falush et al. 2007). We tested whether sympatric species pairs clustered into distinct genotype groups, and determined where putative hybrid genotypes fitted in the genetic structure of the sympatric species pairs. Analysis in Structure used 100,000 discarded burnin runs and a further 1,000,000 MCMC repeats implementing the admixture model. Hypothetical population numbers (K) ranged from 2 to 5, with 10 replicate iterations for each to minimize the inherent stochastic effects of simulations from the algorithm. Output data from Structure v2.3.4 were managed using Structure Harvester online software (Earl and vonHoldt 2012), iterations were averaged using Clumpp v1.1.2 (Jakobsson and Rosenberg 2007) and the results were visualized using Distruct v1.1 (Rosenberg 2004) software. The Greedy Search method implemented in Clumpp was used with random input order and 1,000,000 replications. The search was weighted by the number of individuals in the population as population sample size varied. The averaged K values within each of the datasets were compared using the Evanno method (Evanno et al. 2005) in Structure Harvester online software, to identify the optimal value of K. The dataset with all seven populations represented multiple levels of population structure including several species and populations within species.

We estimated migration rates between the sympatric species using BayesAss v3.0 (Wilson and Rannala 2003). In order to detect introgression rather than potentially infertile F_1_ hybrids we excluded identified F_1_ hybrids. Measurements of migration rate between sympatric populations effectively describes rate of gene flow, rather than organismal movement, as the individuals already share the same geographic space and even daytime refuges. We used a run of 5,000,000 MCMC iterations with a burnin of 1,000,000. Multiple runs with different random starting seeds gave similar results so were not averaged.

## Results

### The frequency of parental species and hybrid phenotypes in sympatry

Similar numbers of each parent species were observed at Kahuterawa (45% *H. thoracica* and 52% *H. crassidens* plus three putative morphological hybrids), and at Mohi (45% *H. thoracica,* 54% *H. trewicki,* and one putative hybrid). In each case, interbreeding parental species would be expected to result in nearly half of all individuals having an intermediate phenotype. Thus, the observed frequencies of putative hybrids deviated significantly from random mating (HW equilibrium) at both locations, (*χ* ^2^ test, *P *<* *0.001). Sixteen percent of the *H. trewicki* sample consisted of adult wētā, while no adult *H. thoracica* were found on the same sampling day at Mohi, suggesting a difference in timing of maturity of these two species (Fisher's exact test, *P* = 0.0038). There was also a significant difference in the number of adults collected where *H. thoracica* and *H. crassidens* are sympatric, but in this case there were more adult *H. thoracica* individuals (31.9% (15/47) compared to 10.9% (6/55); Fisher's exact test, *P *=* *0.00189).

### Phenotype

All specimens that were examined and assigned to one of the three parental types showed the typical combination of color characters for that species (Table 1) except one. This individual from Kahuterawa had a typical *H. crassidens* abdomen but had a pale pronotum more typical of *H. thoracica*. The specimen was assigned to *H. crassidens* based on her prolateral hind tibial spines. Six individuals had intermediate colored abdomens and were identified as putative hybrids (Table 1).

Prolateral tibial spine numbers distinguished *H. thoracica* from the other two species in most cases (Table 1). The allopatric population sample of *H. thoracica* at Taupo included five individuals (23%) with >3 spines, but the allopatric *H. crassidens* population was monomorphic for four spines. Most putative *H. thoracica* (95%) from the Kahuterawa had three prolateral spines on each hind tibia, while most putative *H. crassidens* (91%) had four on each leg. The nine putative hybrid wētā at Kahuterawa resembled either parent species (six or eight spines) or were intermediate with tubercles instead of true spines (Table 1). The single putative hybrid from Mohi had four spines on each leg like *H. trewicki*.

**Figure 2:**
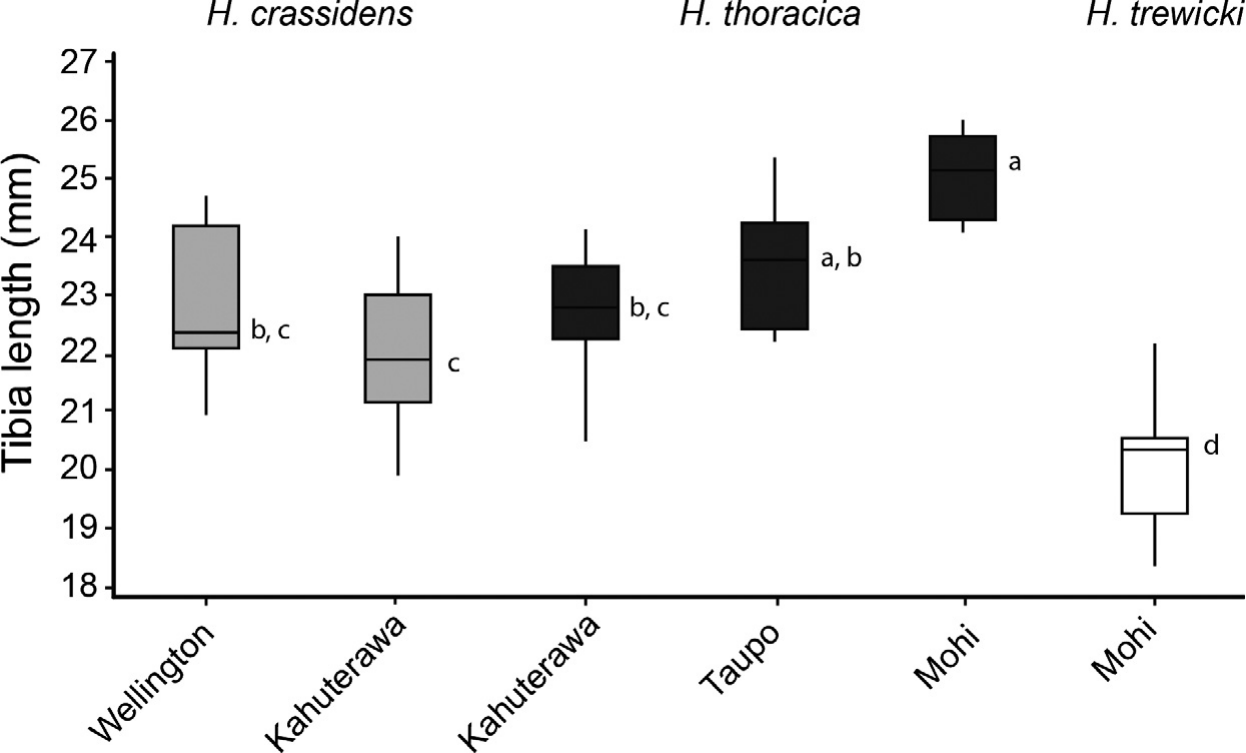
Size variation (hind tibia length) of adult female *Hemideina* sp. collected from six populations. Different letters represent pairs of populations that have means that differ significantly (Tukey's test).

Hind tibia length varied among population samples of adult females, (ANOVA *P *<* *0.001; Fig. 2). Adult female *H. trewicki* had shorter hind tibiae than the other two species (Tukey's test). There was no significant difference in size among the sympatric and allopatric population samples of *H. thoracica* and *H. crassidens* (Fig. 2). Phenotype data are available at http://evolves.massey.ac.nz/Text%20Files/DNA%20Toolkit.htm.

### Cytogenetics

**Figure 3:**
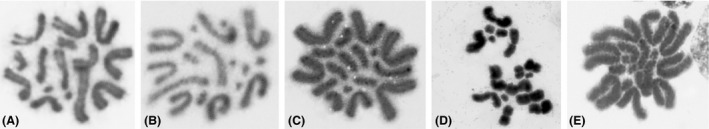
Tree wētā (*Hemideina*) chromosomes as seen at mitosis in males (A) *H. crassidens*, (B) *H. thoracica*, (C) *H. trewicki*. (D) F_1_ hybrid between *H. thoracica* and *H. crassidens*. (E) F_1_ hybrid between *H. thoracica* and *H. trewicki*.

New karyotype data were obtained for 45 wētā (23 Mohi, 22 Kahuterawa; Table 2). All wētā identified morphologically as one of the three parent species had the expected karyotype for that species (Morgan‐Richards 1995; McKean et al. 2015; Fig. 3). No evidence was found of cryptic F_1_ hybrids or unusual karyotypes that would indicate backcross hybrids. In contrast, putative hybrid wētā had the karyotype expected in F_1_ hybrids between their respective parent species (Fig. 3). Female hybrids (*n* = 4) did not provide mitotic cells as they all lacked ovarian material usually used for cytogenetic preparations.

### Mitochondrial DNA sequences

**Figure 4:**
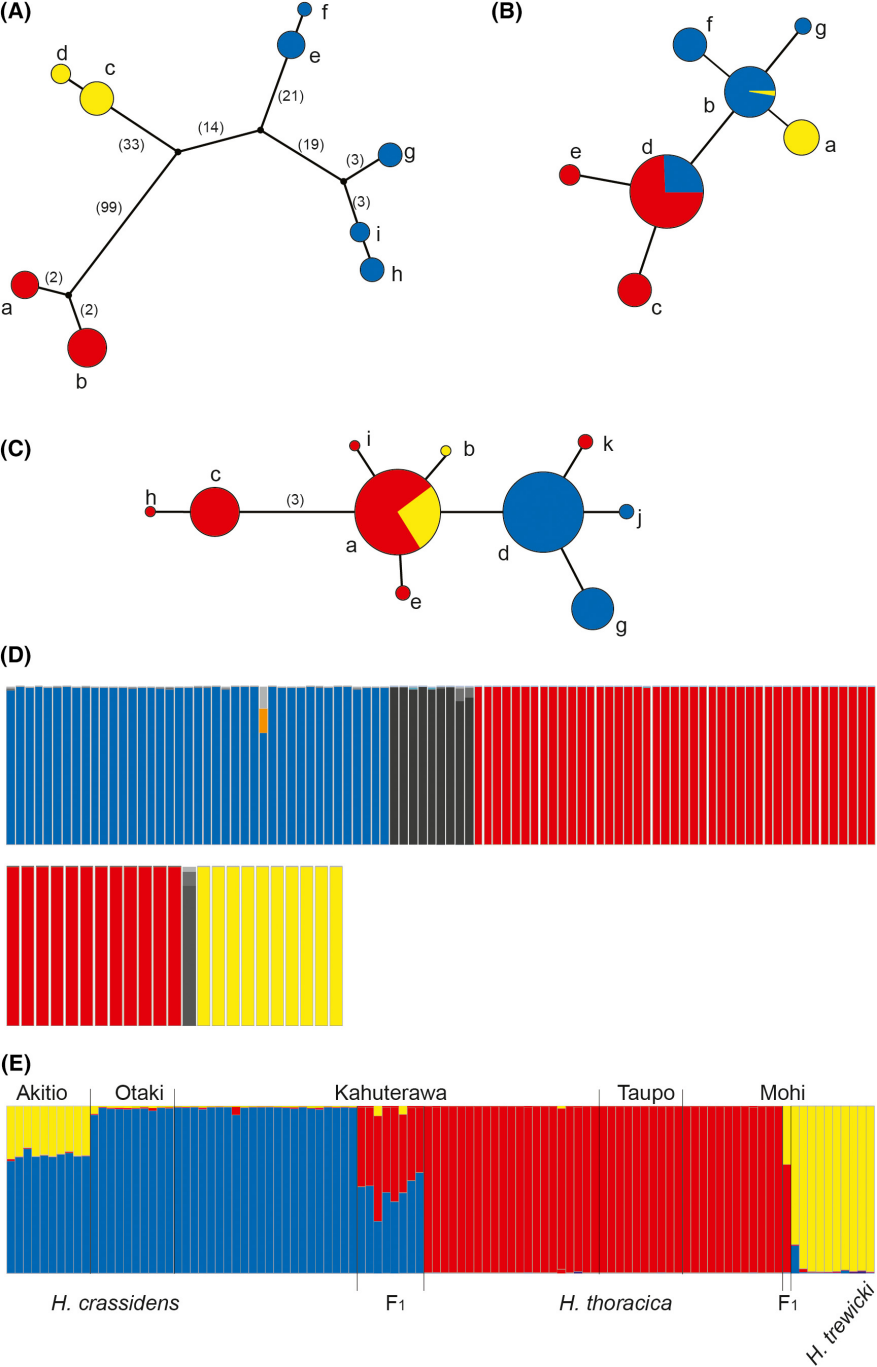
(A) The three North Island tree wētā species (genus *Hemideina*) are polymorphic for a 645 bp sequence from the mtDNA CO1 gene. An integer Neighbor‐Joining network shows inferred relationships of haplotypes. (B) Minimum spanning network showing relationships of seven alleles identified at the nuclear Sperm flagella protein locus. (C): Minimum spanning network shows relationships of 10 Testis kinase‐1 alleles. Colour coded by species of wētā based on phenotype (*H. thoracica* – red; *H. crassidens* – blue; *H. trewicki* – yellow), size of circles scaled by sample size. (D) Probability that individuals belong to a particular parent or hybrid class for the sympatric species pairs (Bayesian inference from NewHybrids) show strong support for the phenotypic classifications of individuals. Top: *Hemideina thoracica* (red) and *H. crassidens* (blue) wētā from Kahuterawa. Bottom: *Hemideina thoracica* (red) and *H. trewicki* (yellow) wētā from Mohi. F_1_ hybrids are indicated in dark gray, B_1_ and B_2_ indicated with pale gray and orange respectively. Bar width is arbitrary. (E) Assignment analyses (using eight nuclear loci) reveals population structure of North Island tree wētā (*K* = 3). Putative hybrids (based on phenotype) are labeled F_1_.

New mitochondrial COI sequences (645 bp) were obtained from 43 wētā and 12 haplotypes were identified (Table 2). These data supplemented previously haplotyped wētā individuals (Bulgarella et al. 2014). Haplotype clusters corresponded with the three species and two distinct *H. crassidens* lineages (Fig. 4A). There was no evidence of mitochondrial haplotype sharing among the three species. Eight out of nine putative *H. thoracica* and *H. crassidens* hybrids had a *H. crassidens* haplotype, from which it can be inferred that they had an *H. crassidens* mother. Only one putative hybrid had a *H. thoracica* mtDNA haplotype. This observation differs from expectations of equal likelihood of the two parent taxa being the mother (Fisher's exact test; *P *=* *0.039). Haplotype data are available http://evolves.massey.ac.nz/Text%20Files/DNA%20Toolkit.htm.

### Nuclear loci

We obtained unambiguous sequences for each locus from 105 wētā. Seven alleles were identified in the trimmed 250 bp alignment of SPEF (Fig. 4B). The common SPEF allele D, was detected in allopatric *H. thoracica* and *H. crassidens* populations indicated it was of shared ancestral origin. In contrast, there was a fixed difference between *H. trewicki* and *H. thoracica*, so SPEF was suitable to differentiate these two species (Table 2). The TESK1 alignment of 269 bp included 10 alleles (Fig. 4C). *Hemideina thoracica* and *H. trewicki* shared the common allele A, however TESK1 differentiates *H. crassidens* from the other two wētā species (Table 2). All putative hybrid wētā were heterozygous at the locus that differentiated their respective parent species (Table 2), supporting the inference that they were hybrids.

No evidence of linkage disequilibrium between any pair of loci was found, nor were there significant deviations from HW expectations in populations that could not be attributed to small population sample size. Genotypes comprising six microsatellite loci were obtained for all 107 wētā except two that lacked data at the HR14 locus. The six microsatellite loci had between 3 and 24 alleles and all loci had alleles that were unique to one species (Table 2).

### Putative F_1_ hybrids

Cytogenetic data confirmed that phenotypic intermediates were interspecific hybrids. Heterozygosity at all nuclear loci that distinguish *H. thoracica* and *H. crassidens* or *H. thoracica* and *H. trewicki* were seen in these phenotypic intermediates (Table 2). All putative hybrids were classified as F_1_ hybrids with at least 0.9 probability using the Bayesian approach implemented in NewHybrids (Fig. 4D). One individual identified as *H. crassidens* based on phenotype had a potentially introgressed allele at one locus and thus a 29% chance of being either a first or second generation backcross (B_1_ or B_2_) between a hybrid and *H. crassidens*. All other individuals were assigned to the species they phenotypically resembled with probability > 0.97.

### Population structure and estimates of introgression

Evidence for three genetically distinct groups was inferred from the Bayesian assignments of individual genotypes from Structure. When assignments were constrained to two or three clusters (*K* = 2 and 3) inferences matched species identification based on phenotype (Fig. 4E). The highest support was found for *K* = 2, at which *H. crassidens* and *H. trewicki* were merged, which is as expected given the closer morphological and phylogenetic similarity of these two species compared with *H. thoracica* (Morgan‐Richards 1995; Trewick and Morgan‐Richards 2004). At *K* = 3 (second highest support) the three species separated. The sympatric populations comprised two species’ genotype clusters as identified by phenotype with low levels of introgression inferred from assignment probabilities (Fig. 4E). Putative hybrids were assigned to either parent population with probabilities of 0.35–0.64, supporting inference of mixed ancestry for putative hybrid individuals (Fig. 4). Genotypes of weta collected from Akitio have assignment probabilities highest for *H.crassidens* (0.77–0.67), the species they match phenotypically. With genotype assignment probabilities of between 0.23–0.33 for *H. trewicki* the weta at Akitio suggest there has been gene flow between these two species, as previously predicted (Morgan‐Richards et al. 2001).

**Figure 5:**
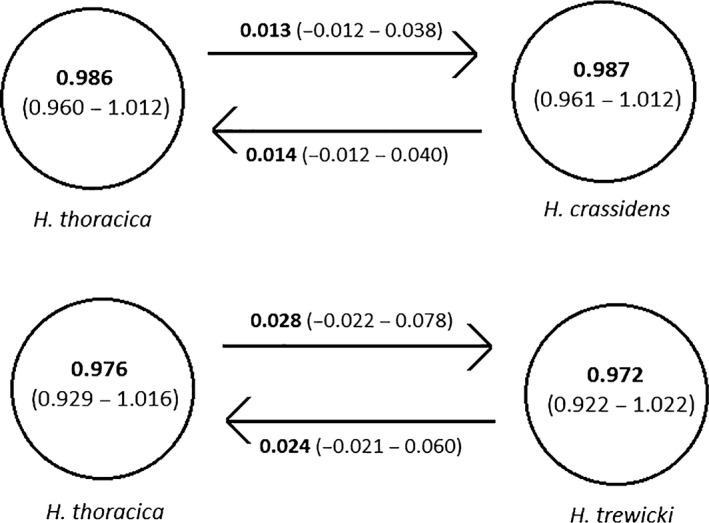
Estimates of gene flow between the sympatric species pairs of tree wētā (*Hemideina* sp.) with 95% confidence intervals using eight polymorphic nuclear markers and Bayesian inference (BayesAss), indicating that gene flow between species is low or nonexistent at both locations.

Population pairwise *F*_ST_ estimates (Table 4) were all greater than zero (*P *<* *0.001). As was expected, population pairs within a species had lower *F*_ST_ values than population pairs between species. Populations in sympatry had substantial differentiation and little allelic exchange; *F*_ST_ = 0.606 (Mohi) and 0.665 (Kahuterawa). The distribution of alleles at two loci provided evidence of potential introgression between species in Kahuterawa (HR12, HR35; Table 2). For example the sample of Kahuterawa *H. thoracica* had two alleles that were also found in *H. crassidens* but were not observed in allopatric population samples of *H. thoracica* (alleles 242, 250; HR35 locus; Table 2). Our estimates of gene flow between the species pairs were very low, and not distinguishable from zero as inferred with BayesAss v3.0 (assuming neutrality; Fig. 5).

**Table 4:** Higher differentiation between species than among populations within species is revealed with pairwise *F*_ST_ estimates for all populations examined in the three North Island tree wētā (genus *Hemideina*), inferred from eight nuclear loci

	*H. crassidens* Akitio	*H.crassidens* Otaki	*H.crassidens* Kahuterawa	*H. thoracica* Kahuterawa	*H. thoracica* Taupo	*H. thoracica* Mohi	*H. trewicki* Mohi
*H. crassidens* Akitio	0						
*H. crassidens* Otaki	0.2871	0					
*H. crassidens* Kahuterawa	0.36865	0.20849	0				
*H. thoracica* Kahuterawa	0.7078	0.6178	0.66525	0			
*H. thoracica* Taupo	0.68443	0.5874	0.64588	0.35945	0		
*H. thoracica* Mohi	0.66953	0.5651	0.63366	0.17132	0.30068	0	
*H. trewicki* Mohi	0.59893	0.46131	0.54326	0.65409	0.70091	0.60617	0

## Discussion

The tree wētā *Hemideina thoracica* meets and mates with two different related species and the long‐term outcome of these two zones of interspecific hybridization will be influenced by the rate of gene flow. Our data shows that at Mohi *H. thoracica* and *H. trewicki* differ in size. *Hemideina thoracica* adult females have longer hind tibia than *H. trewicki,* and longer tibia than conspecifics at Kahuterawa. In addition, the absence of adult *H. thoracica* during sampling at Mohi suggests a difference in developmental timing. Both these traits (size and maturation timing) have the potential to contribute to reproductive isolation but are unlikely to prevent all mating (Gwynne and Jamieson 1998). Confirmation of an F_1_ hybrid collected from the wild demonstrates that these two species will sometimes interbreed where they co‐occur, however, we found no evidence of genetic introgression from karyotype, four nuclear loci, mitochondria, or phenotype. Infertility of F_1_ hybrids might explain a lack of introgression, and this has been suggested for another pair of tree wētā species (*H. femorata* and *H. ricta*; Morgan‐Richards and Townsend 1995). Hybrid sterility is predicted to promote reproductive character displacement.

In contrast, at Kahuterawa we found no evidence of phenotypic divergence in sympatry between *H. thoracica* and *H. crassidens*. Characters that differ are present in both allopatric and sympatric populations. Their similarity in size as adults agrees with other evidence that growth rate (Minards et al. 2014), diet (Dewhurst 2012), and nutritional targets (Wehi et al. 2013a; P. M. Wehi pers. comm.) do not differ significantly between these two species at Kahuterawa. Although all color intermediates in this study were genetically identified as F_1_ hybrids, our analysis with eight loci was not able to distinguish between the hypotheses of low gene flow and no gene flow. However, examination of both the genetic data and spine counts and body color suggest there probably is a low level of gene flow between *H. thoracica* and *H. crassidens*. Spine numbers and pronotum color were not discriminating characters in sympatry but were in the allopatric samples examined here. There is also some allele sharing at two genetic loci in sympatry, and one F_1_ hybrid male mated in captivity with a female *H. thoracica* fathered four offspring (unpubl. data, Mckean, N. E.). Nevertheless, we consider the level of introgression to be low, as fixed differences within our samples were found at two nuclear loci, mitochondrial haplotype and karyotype. The parent species appear to be retaining separate identities in sympatry, so a bimodal hybrid zone appears to be the best description for the contact of *H. thoracica* and *H. crassidens* at Kahuterawa. It is likely these species are differentiated enough to maintain their own evolutionary trajectories in sympatry, but exchange of adaptive alleles cannot be ruled out (Jiggins and Mallet 2000). The similarity of phenotype of *H. thoracica* and *H. crassidens* is likely to result in stronger interspecific competition than between *H. thoracica* and *H. trewicki*. This finding is concordant with the competitive exclusion hypothesis for *H thoracica* and *H. crassidens* based on distribution data, environmental modeling and genetic structure (Bulgarella et al. 2014). *Hemideina thoracica* has probably displaced *H. crassidens* during the current interglacial as the range of *H. thoracica* has expanded south (Trewick and Morgan‐Richards 1995; Bulgarella et al. 2014).

None of the three species of tree wētā appeared to have complete premating barriers to reproduction. Few F_1_ hybrids were detected but this could be due purely to postmating (such as sperm competition) or postzygotic selection (failure to hatch). Adults of *H. thoracica* have been observed in the same daytime refuge cavities as adults of both *H. crassidens* and *H. trewick* (Trewick and Morgan‐Richards 1995, 2000; pers. obs). Even the difference in the timing of maturity between *H. thoracica* and *H. trewicki* is not enough to prevent some first generation hybrids being produced. Bimodal hybrid zones are typically associated with strong pre‐mating barriers (Jiggins and Mallet 2000; and references therein), although the bimodal hybrid zone between two species of chrysomelidae beetles is an exception (Peterson et al. 2005). Further studies involving female mate choice are warranted in order to determine the relative roles of pre‐ and postmating barriers that result in so few hybrids in natural populations. Given the karyotype differences (McKean et al. 2015) chromosomal and other genetic constraints are likely to be involved in limiting F_1_ fertility.

Although the sample of *H. thoracica *× *H. crassidens* hybrids was small (*n* = 9), the significant bias in which species was mother in the production of F_1_ hybrids between *H. thoracica* and *H. crassidens* may indicate that reciprocal crosses are not equally viable. For example, in the sunfish family (Centrarchidae), hybridization in 17 of 18 cases between different species resulted in significantly different viability in F_1_ offspring, depending on which species the mother came from (Bolnick and Near 2005; and references therein). As Dobzansky‐Muller incompatibilities often arise in one species first, they probably have a role in explaining nonreciprocal viability differences (Welch 2004; and references therein). No evidence of *Wolbachia* infections has been detected in this genus (unpubl. data, Morgan‐Richards, M.), another possible source of asymmetrical incompatibilities. It is also possible that postmating prezygotic mechanisms restrict hybridization in an asymmetric fashion, as seen in an example with two *Chyrsochus* beetle species (Monsen et al. 2007), and also in some orthopteran species pairs (e.g. Larson et al. 2012). Future captive breeding experiments could test these hypotheses.

An alternative explanation for the bias in favor of a *H. thoracica* father in most of the F_1_ hybrids is that *H. thoracica* males outcompete *H. crassidens* for mates. Interbreeding results in competition among males for harems of females, as male wētā have a resource‐based polygynous mating system (Kelly 2006; Wehi et al. 2013b). Where sympatric, females of both species will aggregate freely in roost cavities, so any large harem guarded by a male will likely contain both species. This means that any advantage males of one species have to gain and hold a harem will have a significant effect on the relative fitness of both species in sympatry. This form of interspecies competition has been termed ‘sexual exclusion’ (Hochkirch et al. 2007), and may explain how *H. thoracica* has managed to displace *H. crassidens* from much of its former range (Bulgarella et al. 2014). One obvious implication of *H. thoracica* being much larger than *H. trewicki* where they live in sympatry is that *H. thoracica* males may have a strong advantage in defending harems. If this sample is indicative of the population as a whole then early maturing *H. trewicki* will have some opportunity to avoid competition with later maturing *H. thoracica*. This has been seen in other closely related insect species pairs (e.g. Blondheim 1990; Fergus and Shaw 2011). In contrast, *H. thoracica* and *H. crassidens* show no evidence of niche differences where they live in sympatry, and are presumably dealing with strong interspecific competition as are many other hybridizing species (Huxel 1999; and references therein).

## Conflict of Interest

None declared.
